# Severe Pulmonary Hypertension in Critically Ill Children: A 10-Year Single-Center Case Series and Practical Management Considerations

**DOI:** 10.7759/cureus.116689

**Published:** 2026-09-21

**Authors:** Kalliopi Straka, Ioannis Polymerou, Eleni Christakou

**Affiliations:** 1 Pediatric Intensive Care Unit, National and Kapodistrian University of Athens, Athens, GRC; 2 Pediatric Intensive Care Unit-Pediatric Cardiac Intensive Care Unit (PICU-PCICU), Aghia Sophia Children's Hospital, Athens, GRC; 3 Pediatric Cardiology, Mitera Hospital, Athens, GRC; 4 Pediatric Intensive Care Unit-Pediatric Cardiac Intensive Care Unit (PICU-PCICU), Children's Hospital Agia Sophia, Athens, GRC

**Keywords:** mechanical ventilation, pediatric intensive care unit, pediatric pulmonary hypertension, pulmonary hypertensive crisis, right ventricular dysfunction

## Abstract

Severe pulmonary hypertension (PH) in children may decompensate rapidly during intercurrent illness and is associated with substantial morbidity and mortality in the pediatric intensive care unit (PICU). We describe the clinical presentation, intensive care management, and outcomes of four children with severe PH admitted to a tertiary PICU between January 2014 and December 2024.

Three patients were infants, and one was an 11-year-old girl with a history of extreme prematurity and bronchopulmonary dysplasia. All four presented with acute respiratory distress, hypoxemia, and cardiovascular compromise following a recent respiratory tract infection. PH was documented predominantly by echocardiography, with findings including elevated estimated pulmonary pressures, right ventricular (RV) pressure overload, interventricular septal flattening, and/or RV dysfunction. Right heart catheterization was performed in one patient. All patients received supplemental oxygen, diuretics, and sildenafil. Two required invasive mechanical ventilation and vasoactive support, while one patient received additional prostanoid and endothelin receptor antagonist therapy. The median PICU length of stay was 15 days (range: 10-24 days). Two patients survived to hospital discharge, whereas two died following refractory pulmonary hypertensive crises.

These cases highlight respiratory infection as an important potential precipitant of acute decompensation in children with severe PH and illustrate the challenges of supporting the failing RV in the PICU. Although the small sample size and retrospective design preclude conclusions regarding treatment efficacy or prognostic factors, the cases emphasize several practical management priorities: early recognition of deterioration, prompt correction of factors that increase pulmonary vascular resistance, preservation of RV function and systemic perfusion, cautious escalation of respiratory support, and early multidisciplinary involvement. Based on these principles and the published pediatric PH literature, we propose a practical, physiology-based management algorithm for severe PH and pulmonary hypertensive crisis in the PICU.

## Introduction

Pulmonary hypertension (PH) in children comprises a heterogeneous group of disorders characterized by elevated pulmonary arterial pressure and/or pulmonary vascular resistance (PVR), potentially resulting in progressive right ventricular (RV) dysfunction and right heart failure [1,2]. Pediatric PH differs substantially from adult disease because of developmental factors, greater etiologic heterogeneity, and frequent associations with congenital heart disease, developmental lung disease, genetic disorders, and other pediatric-specific conditions [1-8].

Clinical manifestations are often nonspecific and vary according to age. Infants may present with tachypnea, feeding difficulties, hypoxemia, irritability, or failure to thrive, whereas older children may experience exercise intolerance, fatigue, chest pain, or syncope [2,4]. This nonspecific clinical presentation may contribute to delayed recognition and initiation of appropriate treatment.

Acute respiratory infections are particularly important in children with PH. Hypoxemia, hypercapnia, acidosis, inflammation, and increased sympathetic activity may increase PVR, precipitating acute RV dysfunction and pulmonary hypertensive crisis [5,6]. In children with limited RV reserve, this deterioration may progress rapidly to reduced left ventricular filling, systemic hypotension, impaired coronary perfusion, and cardiovascular collapse.

Management in the pediatric intensive care unit (PICU), therefore, requires simultaneous treatment of precipitating factors and preservation of RV function. Particular attention should be directed toward optimizing oxygenation and ventilation, avoiding or promptly correcting factors that increase PVR, maintaining systemic arterial pressure and RV coronary perfusion, and using pulmonary vasodilator and vasoactive therapies when clinically indicated [2,5,6]. Invasive mechanical ventilation warrants particular caution because induction, positive intrathoracic pressure, and changes in preload and RV afterload may precipitate cardiovascular deterioration in patients with compromised RV function. Acute respiratory illnesses may contribute to pulmonary hypertensive decompensation through hypoxemia, hypercapnia, acidosis, inflammation, and altered lung mechanics; however, in children with previously unrecognized pulmonary vascular disease, the reverse relationship is also possible, with limited RV reserve amplifying the clinical severity of an otherwise moderate respiratory illness.

Severe pediatric PH requiring intensive care remains uncommon, and PICU-specific clinical experience is consequently limited. We describe the presentation, intensive care management, and outcomes of four children with severe PH admitted to a tertiary PICU over 10 years. Through these cases, we highlight practical considerations in the recognition and management of acute pulmonary hypertensive decompensation and provide the clinical framework for a proposed PICU management algorithm.

## Case presentation

Study design and setting

We conducted a retrospective descriptive case series in the Pediatric Intensive Care Unit of *Aghia Sophia *Children’s Hospital, a tertiary pediatric referral center in Athens, Greece. Medical records from January 2014 through December 2024 were reviewed to identify children admitted to the PICU with a documented clinical diagnosis of severe PH.

PH had been diagnosed during routine clinical care, predominantly based on echocardiographic assessment. Documented echocardiographic features included elevated estimated pulmonary pressures, RV pressure overload, interventricular septal flattening, and/or RV dysfunction. Right heart catheterization was available in one patient. Because complete invasive hemodynamic characterization and comprehensive etiological investigations were not available for all patients, the cases were not uniformly classified as idiopathic pulmonary arterial hypertension (PAH). For this retrospective series, they are therefore described conservatively as severe pediatric PH with confirmed or incompletely characterized etiology.

Because the study covered 10 years, archived echocardiographic and electrocardiographic source images were only partially retrievable. 

Patients with PH primarily attributable to congenital heart disease or another clearly established secondary cause were not included. Complete invasive hemodynamic characterization was not available for all patients. Accordingly, to avoid retrospective overclassification, the cohort is described conservatively as severe pediatric PH rather than being uniformly classified as idiopathic PAH.

Data extracted from the medical records included demographic characteristics, relevant medical history, preceding respiratory infection, clinical presentation, respiratory and hemodynamic support, echocardiographic findings, PH-directed treatment, PICU length of stay, and clinical outcomes. Where available, additional data included birth and neonatal history, previous cardiopulmonary symptoms, prior cardiac assessment, timing of the initial PH diagnosis, characteristics and severity of the preceding respiratory illness, respiratory support requirements, microbiological or viral investigations, radiographic findings, and follow-up echocardiographic data.

Ethical considerations

The study was conducted in accordance with institutional ethical standards and the principles of the Declaration of Helsinki. Given its retrospective design and the anonymization of patient data, the requirement for informed consent was waived in accordance with institutional policy.

Patient characteristics and clinical course

Four children with severe PH were identified during the study period. Three were infants aged two, three, and seven months, respectively, while the fourth was an 11-year-old girl with a history of extreme prematurity and bronchopulmonary dysplasia. All four patients presented with acute respiratory deterioration characterized by respiratory distress and hypoxemia, with variable degrees of cardiovascular compromise. A recent respiratory illness was documented in all cases. However, because the severity, microbiological etiology, and temporal relationship of the respiratory illness to the pulmonary hypertensive presentation were not uniformly documented, the respiratory illness is considered temporally associated with, rather than definitively causal for, the pulmonary hypertensive deterioration.

In the three infants, PH was first identified during the diagnostic evaluation associated with the current PICU presentation, with review of previous medical records confirming the absence of a prior established diagnosis of PH.

All four patients underwent repeated pediatric cardiology assessments, including echocardiographic evaluation, during their PICU admission. Echocardiography was used to assess PH and RV involvement, while right heart catheterization was additionally performed in one patient. The individual clinical courses are described below, and the principal clinical characteristics, organ-support requirements, PH-directed therapies, and outcomes are summarized in Tables 1 and 2.

The overall clinical course, major cardiopulmonary findings, therapeutic interventions, and outcomes of the four cases are summarized schematically in Figure 1.

**Figure 1:**
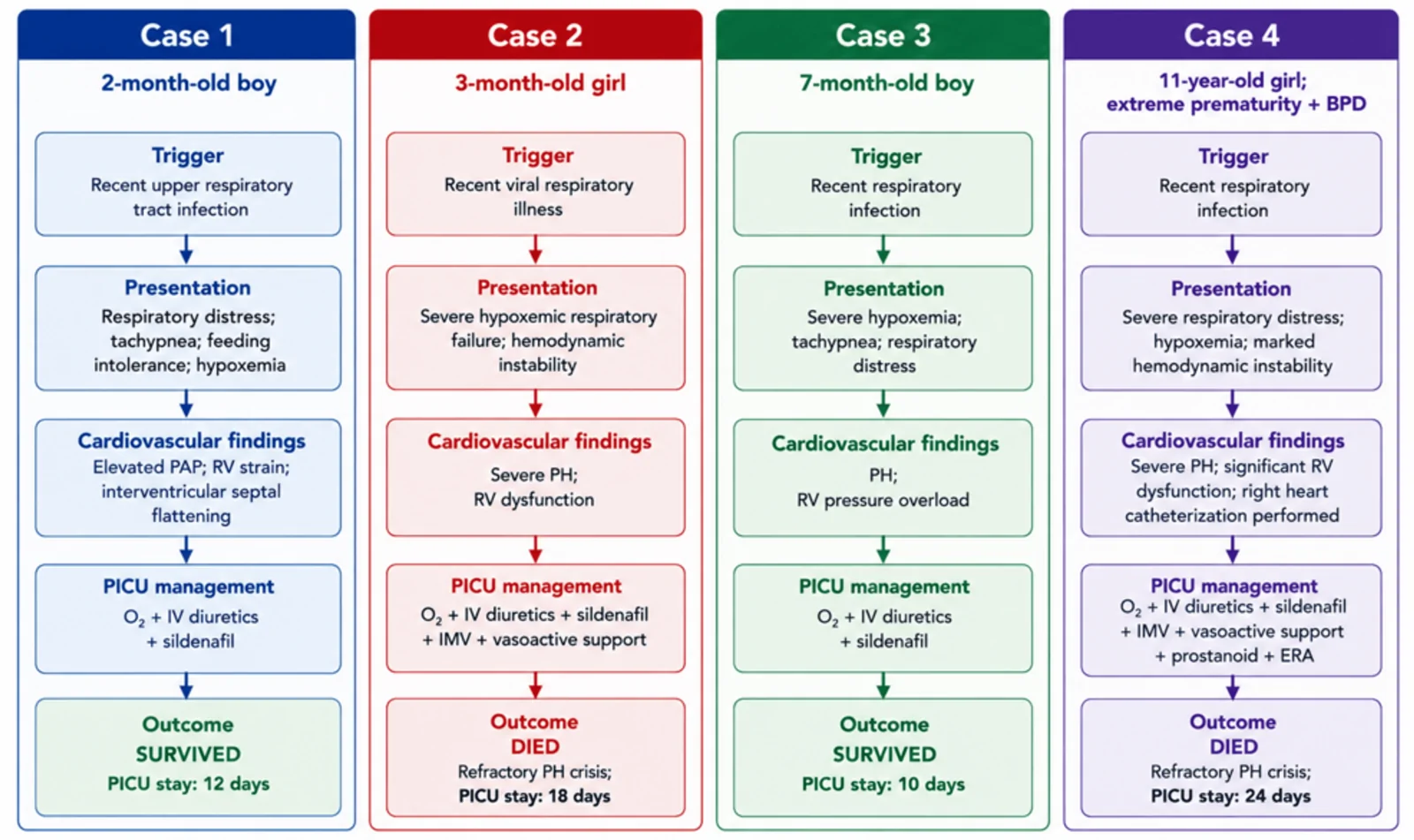
Clinical course, management, and outcomes of the four children with severe PH. Schematic representation of the preceding respiratory illness, clinical presentation, documented cardiovascular findings, major respiratory and hemodynamic interventions, PH-directed therapies, and outcomes of the four patients included in the case series. The figure was created by the authors using Microsoft PowerPoint and is based exclusively on clinical information extracted from the patients’ medical records. It is an original figure and has not been reproduced or adapted from another published source. No generative artificial intelligence was used in its creation. BPD, bronchopulmonary dysplasia; ERA, endothelin receptor antagonist; IMV, invasive mechanical ventilation; IV, intravenous; O₂, supplemental oxygen; PAP, pulmonary arterial pressure; PH, pulmonary hypertension; PICU, pediatric intensive care unit; RV, right ventricular

Case 1

A two-month-old boy was admitted to the PICU following a recent upper respiratory tract infection, presenting with progressive respiratory distress, tachypnea, feeding intolerance, and oxygen desaturation.

He was born at term following an uncomplicated pregnancy and delivery. The neonatal period was unremarkable. He did not require neonatal oxygen therapy, respiratory support, or admission to a neonatal intensive care unit. Before the current illness, no respiratory or cardiovascular symptoms were documented. There was no established diagnosis of PH before the current PICU admission.

Clinical evaluation raised concern for significant cardiopulmonary compromise, prompting pediatric cardiology assessment. Echocardiography documented elevated pulmonary arterial pressures, RV strain, and interventricular septal flattening, consistent with significant RV pressure loading.

The patient was managed with supplemental oxygen and intravenous diuretic therapy, and PH-directed treatment with sildenafil was initiated. Detailed microbiological characterization and objective quantification of the preceding respiratory illness were not available in the retrospective records. His respiratory and cardiovascular status subsequently improved with medical management, and invasive mechanical ventilation or vasoactive support was not required. He survived to hospital discharge after a 12-day PICU stay. His principal clinical features and therapeutic interventions are summarized in Tables 1-2.

Echocardiography documented elevated pulmonary arterial pressures, RV strain, and interventricular septal flattening, consistent with significant RV pressure loading. The echocardiographic appearance of interventricular septal flattening in pediatric PH is shown in Figure 2. 

**Figure 2:**
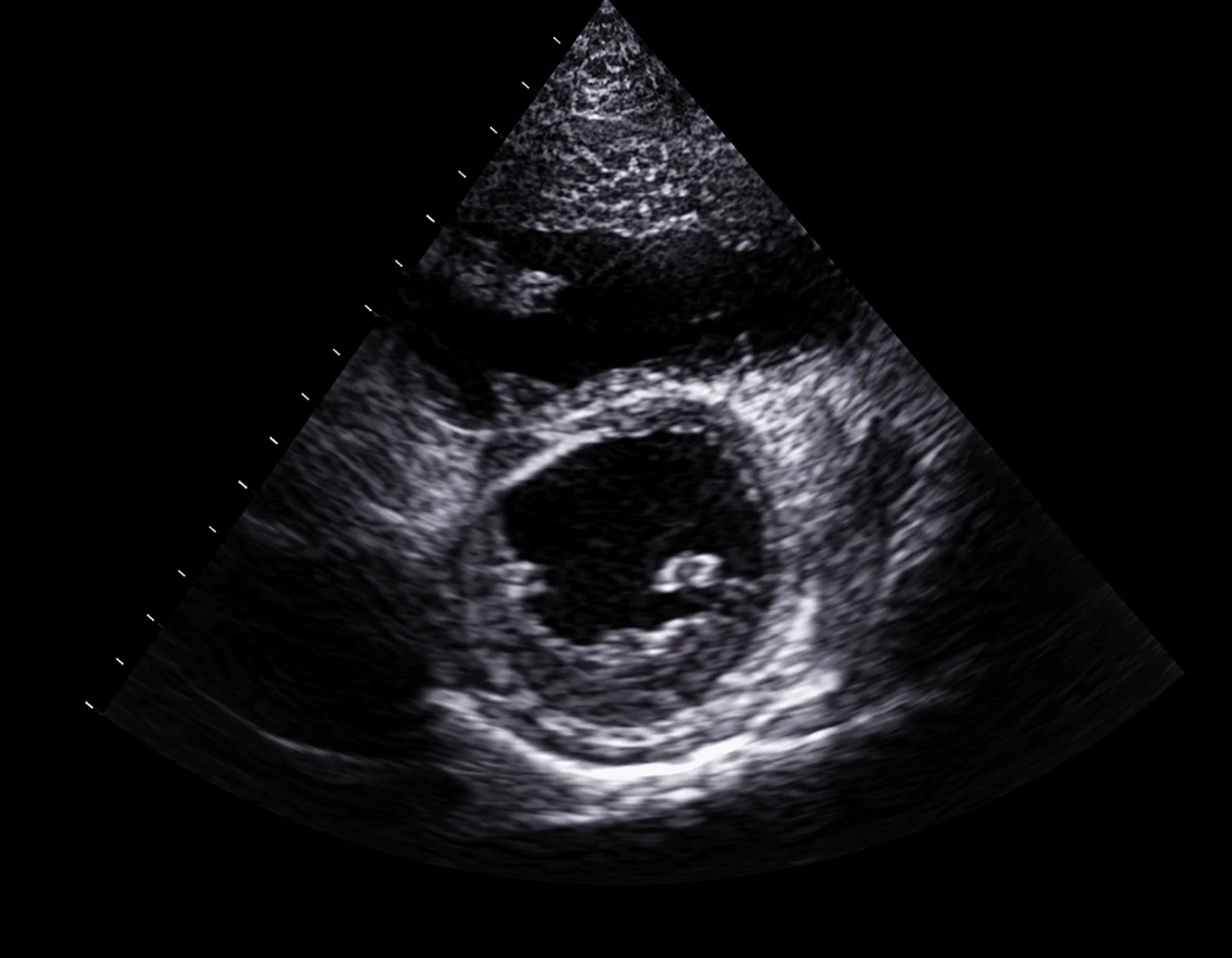
Echocardiographic features of severe pulmonary hypertension. Parasternal short-axis echocardiographic view demonstrating interventricular septal flattening with a D-shaped left ventricular configuration, consistent with significant right ventricular pressure overload.

Case 2

A three-month-old girl was admitted with severe hypoxemic respiratory failure and hemodynamic instability following a recent viral respiratory illness. Echocardiographic evaluation demonstrated severe PH associated with RV dysfunction. Birth and neonatal history were reviewed because severe PH at this age raises the possibility of developmental, parenchymal, congenital, or genetic pulmonary vascular disease. She was born at term, and the neonatal course was unremarkable. Before the current admission, no cardiopulmonary symptoms were documented, and PH had not previously been diagnosed. 

The respiratory illness was characterized by fever, cough, bronchiolitis symptoms, hypoxemia, and radiographic abnormalities.

Because of progressive respiratory and cardiovascular compromise, invasive mechanical ventilation was required together with vasoactive support. Treatment included supplemental oxygen, intravenous diuretics, and PH-directed therapy with sildenafil. Despite correction of potentially reversible contributors to increased PVR, including optimization of oxygenation and ventilation and treatment of the underlying respiratory illness as clinically indicated, severe PH and RV deterioration persisted. Despite intensive respiratory, hemodynamic, and pulmonary vasodilator support, the patient's condition progressed to a refractory pulmonary hypertensive crisis with worsening cardiovascular failure. Sustained hemodynamic stabilization could not be achieved, and the patient died during the PICU admission after an 18-day stay. Her clinical features, organ-support requirements, and PH-directed treatment are summarized in Tables 1-2. The echocardiographic findings are shown in Figure 3.

**Figure 3:**
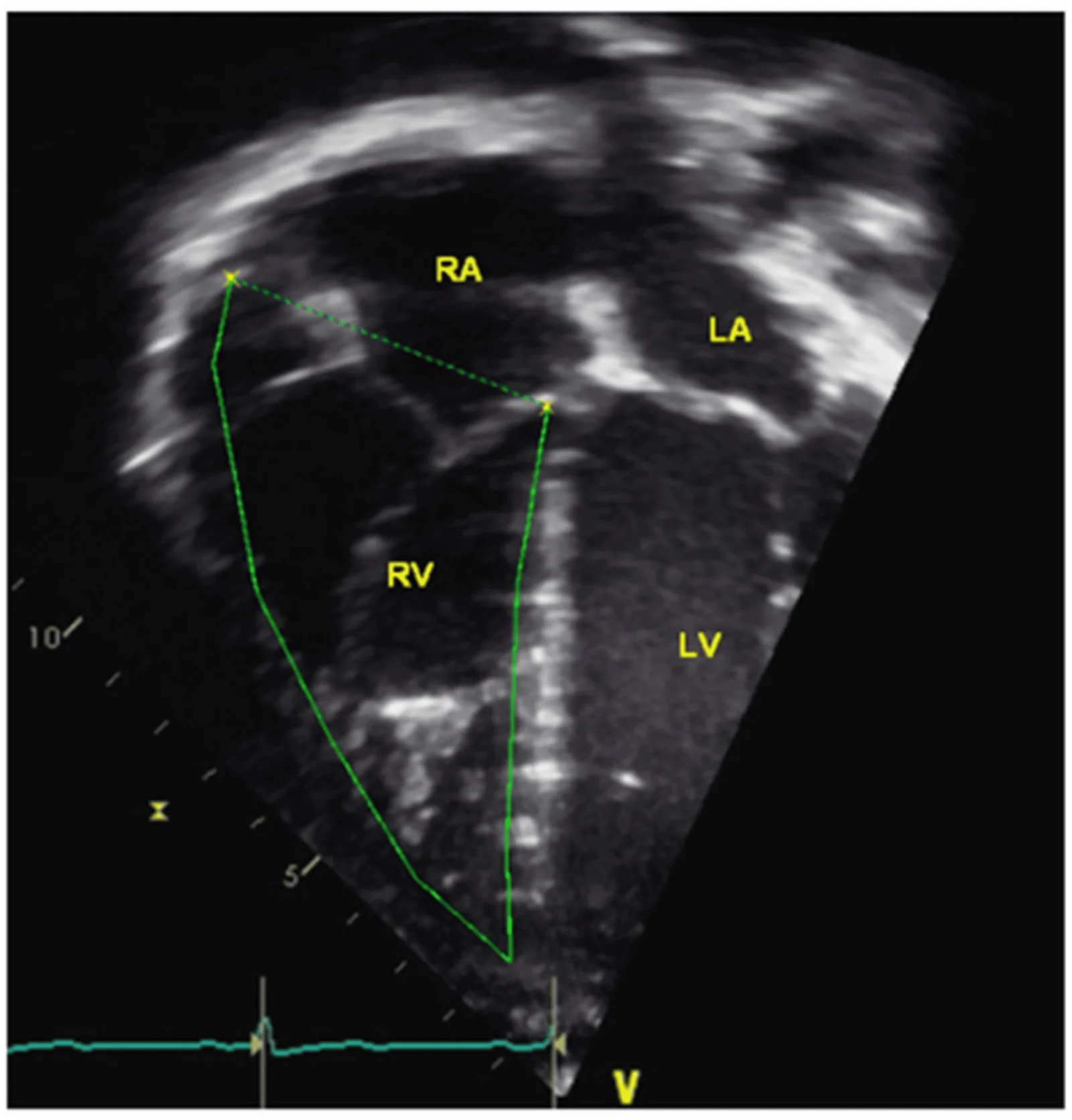
Echocardiographic assessment of right ventricular enlargement. Apical four-chamber echocardiographic view demonstrating assessment of right ventricular dimensions and enlargement, illustrating the pattern of right ventricular involvement documented in Case 2. RA, right atrium; RV, right ventricle; LA, left atrium; LV, left ventricle

Case 3

A seven-month-old boy presented following a recent respiratory tract infection with severe hypoxemia, tachypnea, and respiratory distress requiring PICU admission. Pediatric cardiology assessment and echocardiography demonstrated PH with evidence of RV pressure overload. 

Birth, neonatal, and pre-PICU history were reviewed, including previous respiratory symptoms, feeding difficulties, poor weight gain, recurrent respiratory infections, previous hospitalizations, oxygen requirement, and previous cardiac investigations. The respiratory illness may have increased PVR or unmasked previously compensated pulmonary vascular disease.

The patient was treated with supplemental oxygen, intravenous diuretics, and sildenafil. His respiratory and cardiovascular condition subsequently stabilized with medical management, without the need for invasive mechanical ventilation or vasoactive support. He survived to hospital discharge following a 10-day PICU stay. His clinical presentation, treatment, and outcome are summarized in Tables 1-2. The echocardiographic findings are shown in Figure 4.

**Figure 4:**
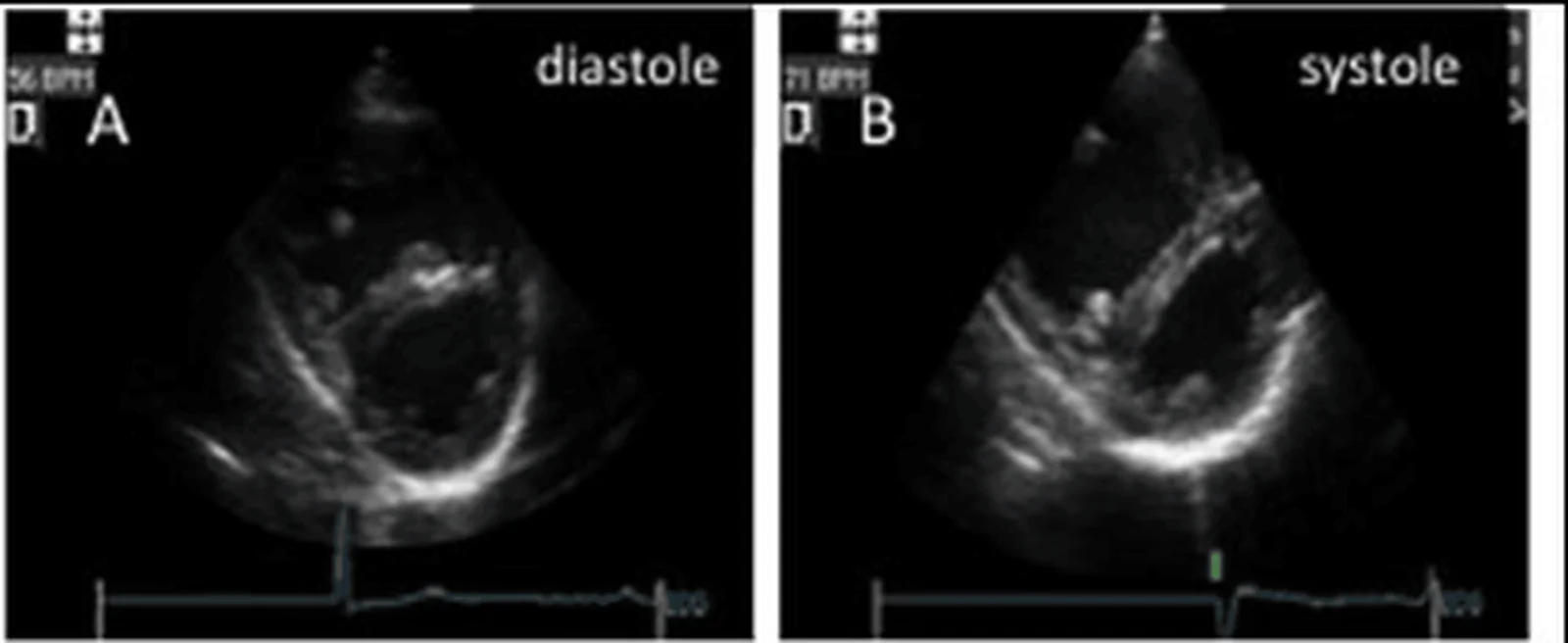
Echocardiographic features of right ventricular pressure overload in pediatric pulmonary hypertension. Parasternal short-axis echocardiographic views demonstrating interventricular septal configuration during (A) diastole and (B) systole. Systolic septal flattening and alteration of left ventricular geometry illustrate the echocardiographic features of right ventricular pressure overload documented in Case 3.

Case 4

An 11-year-old girl with a history of extreme prematurity and bronchopulmonary dysplasia was admitted following a respiratory tract infection with severe respiratory distress, hypoxemia, and marked hemodynamic instability. Echocardiographic evaluation documented severe PH with significant RV dysfunction. Given the history of extreme prematurity and bronchopulmonary dysplasia, developmental lung disease was considered a plausible contributor to the PH. Accordingly, this case was not classified as idiopathic PAH. Previous echocardiographic examinations demonstrated evidence of PH before the current admission.

Her clinical condition deteriorated despite initial supportive and PH-directed treatment, necessitating invasive mechanical ventilation and vasoactive support. Echocardiographic evaluation documented severe PH with significant RV dysfunction, indicating advanced right-sided pressure load and impaired RV performance. Because of persistent clinical deterioration and the severity of the pulmonary vascular disease, right heart catheterization was subsequently performed for further hemodynamic characterization and to guide therapeutic escalation.

PH-directed therapy was escalated beyond sildenafil to include prostanoid therapy and an endothelin receptor antagonist. Despite advanced respiratory and hemodynamic support and escalation of pulmonary vasodilator treatment, she developed a refractory pulmonary hypertensive crisis with progressive hemodynamic failure and died during the PICU admission after a 24-day stay.

Given her history of extreme prematurity and bronchopulmonary dysplasia, pulmonary vascular disease associated with developmental lung disease may have contributed to the PH phenotype. Consequently, this patient cannot be retrospectively classified with certainty as having idiopathic PAH. Her clinical course, organ-support requirements, and PH-directed therapies are summarized in Tables 1-2. 

Cardiovascular evaluation demonstrated features of severe right-sided pressure overload, while echocardiography documented severe PH with significant RV dilatation and dysfunction, indicating impaired RV performance (Figure 5).

**Figure 5:**
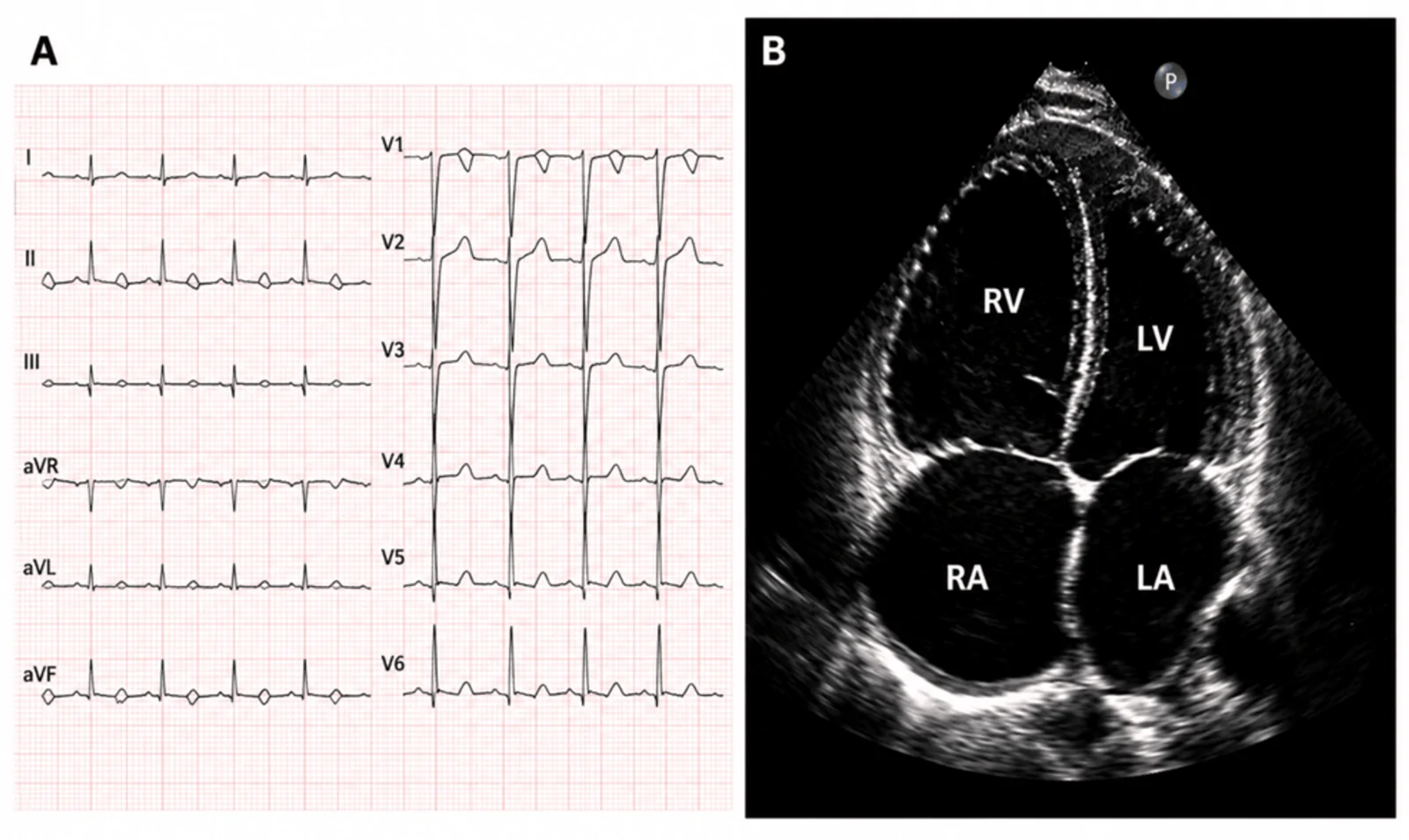
Cardiovascular findings associated with advanced pediatric pulmonary hypertension in the patient. (A) Twelve-lead electrocardiogram obtained during clinical evaluation. (B) Apical four-chamber echocardiographic view demonstrating marked right atrial and right ventricular (RV) enlargement with evidence of RV pressure overload and impaired RV performance.

Results

The principal clinical characteristics and outcomes of the four patients are summarized in Table 1.

**Table 1:** Clinical characteristics and outcomes of pediatric patients with PH. PH, pulmonary hypertension; RV, right ventricular

Patient	Age/Sex	Relevant history	Preceding respiratory infection	Clinical presentation	Documented echocardiographic/hemodynamic findings
1	2 months/M	None recorded	Yes	Progressive respiratory distress, tachypnea, feeding intolerance, hypoxemia	Elevated pulmonary arterial pressures, RV strain, interventricular septal flattening
2	3 months/F	None recorded	Yes	Severe hypoxemic respiratory failure, hemodynamic instability	Severe PH with RV dysfunction
3	7 months/M	None recorded	Yes	Severe hypoxemia, tachypnea, respiratory distress	PH with RV pressure overload
4	11 years/F	Extreme prematurity; bronchopulmonary dysplasia	Yes	Severe respiratory distress, hypoxemia, marked hemodynamic instability	Severe PH with significant RV dysfunction; right heart catheterization performed

Respiratory, hemodynamic, and PH-directed therapies are summarized in Table 2.

**Table 2:** Respiratory, hemodynamic, and PH-directed treatment. IV, intravenous; PH, pulmonary hypertension; PICU, pediatric intensive care unit

Patient	Oxygen	Invasive mechanical ventilation	IV diuretics	Sildenafil	Additional PH-directed therapy	Vasoactive support	PICU stay	Outcome
1	Yes	No	Yes	Yes	None	No	12 days	Survived
2	Yes	Yes	Yes	Yes	None	Yes	18 days	Died
3	Yes	No	Yes	Yes	None	No	10 days	Survived
4	Yes	Yes	Yes	Yes	Prostanoid; endothelin receptor antagonist	Yes	24 days	Died

The median PICU length of stay was 15 days (range: 10-24 days). All four patients received supplemental oxygen, intravenous diuretics, and sildenafil. Two patients required invasive mechanical ventilation and vasoactive support; both subsequently died following refractory pulmonary hypertensive crises. One of these patients required escalation of PH-directed therapy with a prostanoid and an endothelin receptor antagonist and underwent right heart catheterization. The remaining two patients were managed without invasive mechanical ventilation or vasoactive support and survived to hospital discharge. Overall mortality in this small cohort was 50%. Management also focused on potentially reversible factors capable of increasing PVR, including correction of hypoxemia, optimization of ventilation, avoidance or correction of hypercapnia and acidosis, and careful fluid management. Treatment of the associated respiratory illness was provided according to the clinical and microbiological findings available for each patient.

Given the very small sample size and differences in disease severity, these observations should not be interpreted as evidence that invasive mechanical ventilation or vasoactive treatment independently increases mortality. Rather, the requirement for advanced organ support most likely reflects greater severity of cardiopulmonary decompensation.

## Discussion

This case series illustrates the clinical complexity of severe PH in critically ill children and the potential for rapid progression from respiratory deterioration to RV dysfunction and cardiovascular collapse.

All four patients presented with acute respiratory illness associated with hypoxemia and cardiopulmonary deterioration. Respiratory disease may increase PVR through hypoxemia, hypercapnia, acidosis, inflammation, sympathetic activation, and adverse alterations in lung mechanics. Nevertheless, the temporal association observed in this series does not establish causality. Acute respiratory illness may have precipitated or amplified PH and RV decompensation in patients with pre-existing pulmonary vascular disease; conversely, previously unrecognized severe PH and limited RV reserve may have increased the clinical severity of an otherwise less severe respiratory illness. This distinction is particularly important in infants, in whom acute bronchiolitis and other parenchymal respiratory diseases may themselves be associated with transient elevations in pulmonary artery pressure. Consequently, the relative contributions of acute respiratory disease and pre-existing pulmonary vascular abnormalities could not be reliably distinguished in the present retrospective series.

Given the small size and descriptive nature of the cohort, these observations cannot establish prognostic associations; rather, they highlight the importance of early recognition of decompensation, assessment and protection of RV function, and careful escalation of respiratory and hemodynamic support.

A preceding respiratory tract infection was documented in all four patients. Respiratory infections may precipitate pulmonary hypertensive decompensation through several interacting mechanisms, including hypoxemia, hypercapnia, acidosis, inflammation, and increased sympathetic activity, all of which may contribute to increased PVR [5-7]. In a child with limited RV reserve, an acute increase in PVR increases RV afterload and may lead to RV dilatation and dysfunction. Progressive RV failure may subsequently impair left ventricular filling, reduce systemic cardiac output and coronary perfusion, and ultimately result in cardiovascular collapse.

Severe PH presenting during early infancy warrants a broad etiological differential. In addition to idiopathic or heritable PAH, potential causes include developmental and parenchymal lung disease, pulmonary hypoplasia, pulmonary venous abnormalities, congenital cardiac disease, hypoxia-associated PH, and genetic disorders affecting pulmonary vascular or lung development. Importantly, the absence of an immediately identifiable secondary cause does not establish idiopathic PAH. In the present cohort, comprehensive invasive, genetic, and developmental lung evaluation was not uniformly available; therefore, the precise etiology of PH in the infant cases remains incompletely characterized.

This pathophysiological sequence has important implications for intensive care management. Respiratory deterioration in a child with known or suspected severe PH should not be considered solely a pulmonary problem. Worsening hypoxemia, systemic hypotension, poor peripheral perfusion, altered mental status, or other evidence of low cardiac output should raise concern for evolving RV failure and pulmonary hypertensive crisis. Early recognition of this transition may provide an opportunity to intervene before irreversible circulatory collapse occurs.

Echocardiography and hemodynamic assessment

Echocardiography plays a central role in the noninvasive assessment of suspected pediatric PH and provides clinically important information regarding estimated pulmonary pressures, RV pressure loading, interventricular septal configuration, and RV function [1,2]. In the critically ill child, serial echocardiographic assessment may also assist in evaluating changes in RV performance and ventricular interaction during clinical deterioration and treatment.

In this series, diagnosis and assessment of disease severity during clinical care were predominantly based on echocardiography. Right heart catheterization, which remains important for definitive hemodynamic characterization and classification of PAH, was performed in only one case [1,2]. An important distinction must therefore be made between clinically documented severe PH and definitively characterized idiopathic PAH. The retrospective nature of this series and incomplete invasive hemodynamic data do not permit uniform retrospective confirmation of PH subtype in all four patients. This is particularly relevant to Case 4, whose history of extreme prematurity and bronchopulmonary dysplasia raises the possibility of pulmonary vascular disease associated with developmental lung disease. Idiopathic PAH in children remains a particularly challenging condition because of its rarity, heterogeneous presentation, and potentially severe clinical course [9,10].

The inability to retrieve all archived echocardiographic source images represents an additional limitation. Although this does not negate the contemporaneously documented echocardiographic diagnoses, it prevents standardized retrospective reassessment of disease severity and direct comparison of quantitative echocardiographic parameters among patients.

In infants and children presenting with severe respiratory distress, particularly when hypoxemia, hemodynamic compromise, or deterioration is disproportionate to the apparent pulmonary disease or persists despite appropriate respiratory support, concomitant PH and RV dysfunction should be considered. Early echocardiographic assessment may identify elevated pulmonary pressures, RV pressure loading, interventricular septal abnormalities, and RV dysfunction and may therefore influence subsequent management.

Pulmonary hypertensive crisis and protection of the RV

Acute management of severe PH in the PICU requires simultaneous treatment of precipitating factors and protection of RV function. Hypoxemia, hypercapnia, acidosis, pain, agitation, and excessive airway pressures should be avoided or promptly corrected because they may further increase PVR [5,6]. Adequate systemic arterial pressure is equally important, as systemic hypotension may compromise RV coronary perfusion and accelerate the transition from compensated RV dysfunction to circulatory failure.

Volume status requires careful and repeated assessment. Both inadequate preload and excessive volume loading may impair cardiac output in severe RV dysfunction. Diuretic therapy may therefore be appropriate in patients with evidence of venous congestion or volume overload, but fluid and diuretic management should be individualized according to the patient's clinical and hemodynamic status [5,11,12].

All four patients in this series received supplemental oxygen, diuretics, and sildenafil. Sildenafil, a phosphodiesterase-5 inhibitor, promotes pulmonary vasodilation through enhancement of cyclic guanosine monophosphate signaling and is widely used in pediatric PAH [11-13]. The most severely affected patient received escalation to prostanoid therapy and an endothelin receptor antagonist in addition to sildenafil. Combination pulmonary vasodilator therapy may be appropriate in selected children with severe or progressive PAH; however, treatment should be individualized according to the underlying PH phenotype, clinical severity, hemodynamic status, and specialist assessment [1,2].

In an acute pulmonary hypertensive crisis or acute right-sided heart failure secondary to increased PVR, rapidly acting selective pulmonary vasodilation should be considered. Current pediatric resuscitation guidelines recommend inhaled nitric oxide or prostacyclin as initial pulmonary vasodilator therapy in this setting [5,14,15]. The choice and subsequent escalation of PH-directed therapy should be individualized with specialist input, taking into account the underlying PH phenotype, systemic blood pressure, RV function, and response to initial treatment.

The present series cannot determine the effectiveness of any individual therapy because of its small sample size, heterogeneous clinical severity, individualized treatment, and absence of a comparator group. The therapeutic interventions reported here should therefore be interpreted descriptively rather than as evidence of comparative efficacy.

Mechanical ventilation and hemodynamic support

Two patients required invasive mechanical ventilation because of severe respiratory failure and cardiovascular instability. Mechanical ventilation in severe PH presents a particular challenge. Positive intrathoracic pressure may reduce systemic venous return and alter RV preload, while lung overdistension, excessive airway pressures, hypoxemia, hypercapnia, and acidosis may further increase PVR and RV afterload [5,7,10].

The peri-intubation period may be especially hazardous in a child with compromised RV function. Loss of spontaneous breathing, induction-related systemic vasodilation, reduced venous return, and abrupt changes in intrathoracic pressure may precipitate acute hemodynamic deterioration. When invasive ventilation is unavoidable, intubation should therefore be considered a high-risk hemodynamic intervention. Careful preparation, optimization of systemic perfusion before induction, avoidance of systemic hypotension, prevention of hypoxemia, hypercapnia, and acidosis, and limitation of unnecessarily high airway pressures are important components of management.

Vasoactive and inotropic support may be required to maintain systemic arterial pressure, preserve RV coronary perfusion, and support cardiac output in patients with acute RV failure. Selection of vasoactive therapy should be individualized according to systemic blood pressure, ventricular function, and the underlying hemodynamic profile, with the overall objective of maintaining adequate systemic perfusion without further increasing RV afterload.

Both patients who required invasive mechanical ventilation and vasoactive support died, whereas the two cases who did not require these interventions survived. This observation must be interpreted cautiously and should not be considered evidence of a causal relationship between mechanical ventilation, vasoactive therapy, and mortality. The requirement for advanced cardiopulmonary support more plausibly reflects greater disease severity and advanced RV compromise. Previous pediatric studies have similarly demonstrated substantial morbidity and mortality among critically ill children with PH [7,13,14].

Practical approach to acute decompensation in the PICU

The cases in this series highlight several recurring priorities in the management of acute pediatric PH decompensation. The first is rapid recognition of a potential pulmonary hypertensive crisis and identification of reversible precipitating factors, particularly respiratory infection, hypoxemia, hypercapnia, acidosis, pain, and agitation. The second is early assessment of RV pressure loading and function, predominantly by echocardiography. The third is preservation of systemic perfusion and RV coronary perfusion through careful management of preload and appropriate vasoactive or inotropic support when required.

Respiratory support should be escalated cautiously. Avoidance of invasive mechanical ventilation may be desirable when adequate oxygenation, ventilation, and clinical stability can be safely maintained without intubation; however, necessary intubation should not be delayed in progressive respiratory failure. When invasive ventilation is required, the procedure should be regarded as a high-risk cardiopulmonary intervention and performed with appropriate expertise, preparation, and hemodynamic support.

PH-directed therapy should be individualized according to the underlying PH phenotype and severity of decompensation, with early involvement of clinicians experienced in pediatric PH. Children with refractory pulmonary hypertensive crisis, progressive RV failure, or persistent low cardiac output despite optimized conventional therapy require rapid multidisciplinary reassessment and consideration of advanced rescue strategies in an appropriately experienced center.

Management should simultaneously address both the pulmonary vascular crisis and potentially reversible precipitating factors. Correction of hypoxemia, hypercapnia, and acidosis, optimization of lung recruitment and airway pressures, treatment of underlying respiratory disease when identified, avoidance of excessive fluid loading, and maintenance of adequate systemic perfusion are central components of acute management.

Based on these principles, together with the clinical experience represented by this case series and the published pediatric PH literature, we propose a practical management algorithm for children presenting to the PICU with severe PH or pulmonary hypertensive crisis (Figure 6). The algorithm emphasizes early recognition and correction of reversible triggers, echocardiographic assessment, preservation of RV function and systemic perfusion, cautious escalation of respiratory support, appropriate PH-directed therapy, and early multidisciplinary escalation in refractory disease. Children with refractory pulmonary hypertensive crisis, progressive RV failure, or persistent low cardiac output despite optimized conventional therapy require rapid multidisciplinary reassessment and consideration of advanced rescue strategies in an appropriately experienced center. In refractory PH associated with low cardiac output or profound respiratory failure despite optimal medical therapy, extracorporeal membrane oxygenation may be considered as a rescue strategy in appropriately selected patients [15].

**Figure 6:**
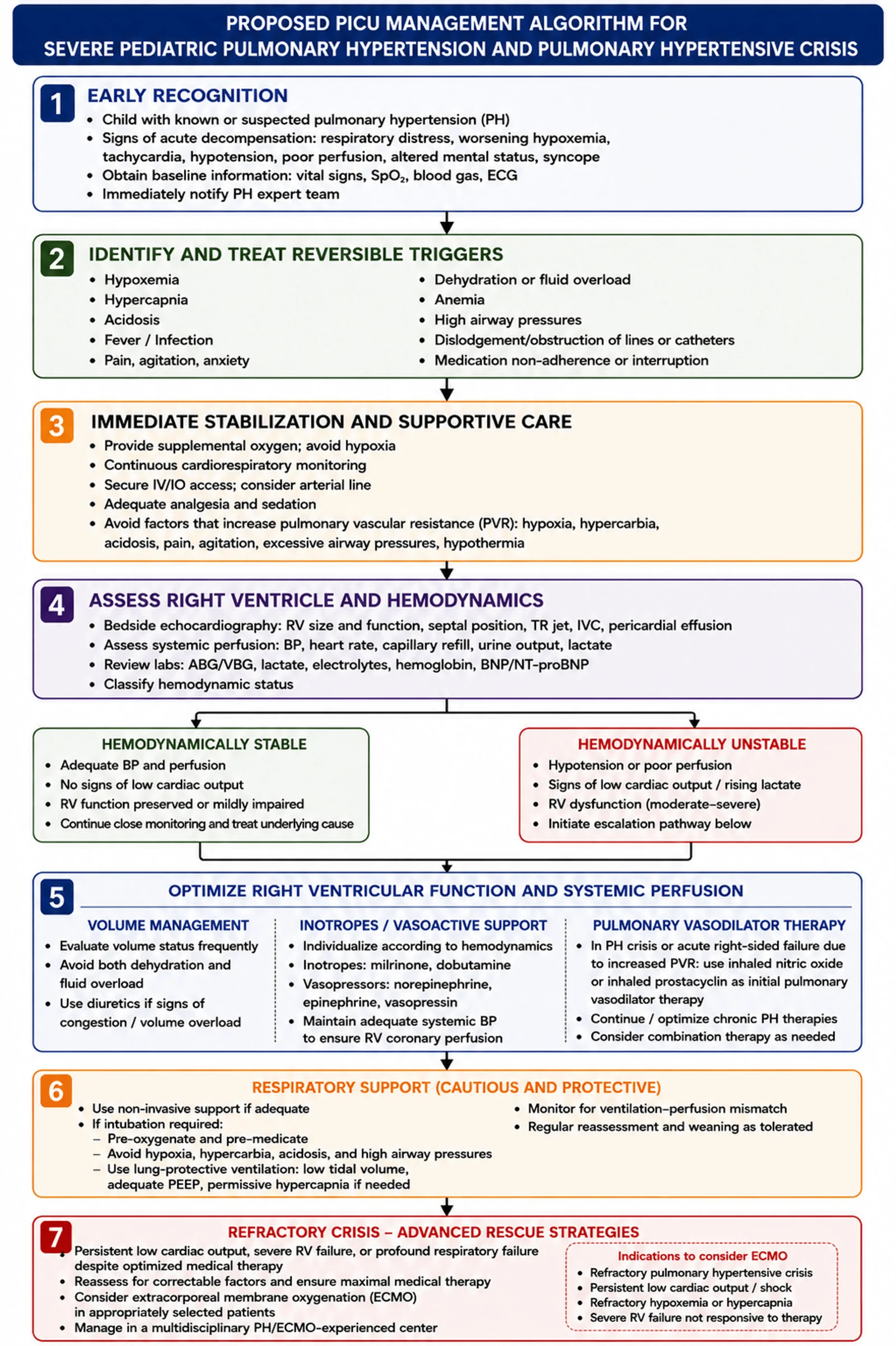
Proposed PICU management algorithm for severe pediatric PH and pulmonary hypertensive crisis. The proposed algorithm provides a physiology-based framework for the recognition and management of severe PH and pulmonary hypertensive crisis in critically ill children. Management priorities include prompt identification and correction of reversible triggers, assessment and protection of RV function, maintenance of systemic perfusion, appropriate pulmonary vasodilator therapy, cautious escalation of respiratory support, and early multidisciplinary management. In pulmonary hypertensive crisis or acute right-sided heart failure secondary to increased PVR, inhaled nitric oxide or inhaled prostacyclin may be considered as initial selective pulmonary vasodilator therapy [15]. In refractory PH associated with low cardiac output or profound respiratory failure despite optimized medical therapy, ECMO may be considered in appropriately selected patients [15]. This figure is an original creation by the authors, developed using Microsoft Word. It has not been reproduced or adapted from any previously published source and was not generated using artificial intelligence. ECMO, extracorporeal membrane oxygenation; PH, pulmonary hypertension; PICU, pediatric intensive care unit; PVR, pulmonary vascular resistance; RV, right ventricular

Limitations

This study has several limitations. First, it represents a retrospective, single-center experience and includes only four patients, reflecting the rarity of severe pediatric PH requiring admission to the PICU. The small sample size precludes meaningful statistical analysis and limits the generalizability of the observations.

Second, definitive hemodynamic characterization by right heart catheterization was available in only one case. The precise etiologic classification of PH therefore cannot be retrospectively confirmed in every case, and the cohort should not be interpreted as a homogeneous series of confirmed idiopathic PAH. This limitation is particularly relevant to the patient with a history of extreme prematurity and bronchopulmonary dysplasia, in whom pulmonary vascular disease associated with developmental lung disease may have contributed to the PH phenotype.

Third, because the study covered 10 years, only a few archived echocardiographic and electrocardiographic images were consistently retrievable. 

Fourth, treatment was individualized according to clinical severity and evolved over the study period. The small sample size, heterogeneity of clinical presentation, and absence of a comparator group preclude assessment of treatment efficacy or identification of independent prognostic factors. In particular, the observation that both patients requiring invasive mechanical ventilation and vasoactive support died should be interpreted as a marker of greater disease severity rather than evidence of a causal relationship between these interventions and mortality.

Additional limitations relate to incomplete historical characterization. Detailed perinatal and neonatal records, pre-PICU cardiopulmonary symptoms, prior echocardiographic findings, microbiological characterization of respiratory illnesses, and objective measures of respiratory infection severity were not uniformly available across the 10-year study period. Consequently, the temporal direction between respiratory illness and pulmonary hypertensive/RV deterioration cannot be determined. Furthermore, because comprehensive etiological testing, invasive hemodynamic assessment, and genetic evaluation were not performed uniformly, the infant cases cannot be reliably classified as idiopathic PAH, and alternative developmental, pulmonary, vascular, or genetic etiologies cannot be excluded.

Despite these limitations, this case series provides clinically relevant observations regarding a rare but potentially catastrophic presentation in the PICU. The shared pattern of respiratory infection preceding acute deterioration and the challenges encountered in supporting RV function and systemic perfusion highlight practical priorities that may assist clinicians managing children with severe PH or pulmonary hypertensive crisis.

## Conclusions

Severe PH in children may decompensate rapidly during intercurrent respiratory illness and represents a challenging cardiopulmonary emergency in the PICU. Early recognition of evolving RV dysfunction, prompt identification and correction of factors that increase pulmonary vascular resistance, careful optimization of volume status, preservation of systemic arterial pressure and RV coronary perfusion, and timely specialist-guided PH therapy are central components of management.

Respiratory support should be individualized according to clinical severity. Although invasive mechanical ventilation may be unavoidable in severe respiratory failure, it should be approached as a high-risk cardiopulmonary intervention in children with significant PH and RV dysfunction, with particular attention to peri-intubation hemodynamic stability and avoidance of hypoxemia, hypercapnia, acidosis, systemic hypotension, and excessive airway pressures.

In this four-patient series, two children survived to hospital discharge, whereas two with severe cardiopulmonary decompensation died following refractory pulmonary hypertensive crises. Given the small sample size and retrospective design, these outcomes cannot establish prognostic factors or comparative treatment effects. Nevertheless, the cases illustrate the importance of a structured, physiology-based approach centered on early recognition, treatment of reversible triggers, protection of RV function, maintenance of systemic perfusion, cautious escalation of respiratory support, and early multidisciplinary involvement.

The proposed PICU management algorithm integrates these principles into a practical clinical framework for the initial assessment and escalation of care in children with severe PH or pulmonary hypertensive crisis. Further multicenter studies and standardized pediatric PH registries are needed to better define risk factors, optimize intensive care strategies, and improve outcomes in this vulnerable population.
